# Effect of Combination Therapy on Joint Destruction in Rheumatoid Arthritis: A Network Meta-Analysis of Randomized Controlled Trials

**DOI:** 10.1371/journal.pone.0106408

**Published:** 2014-09-22

**Authors:** Niels Graudal, Thorbjørn Hubeck-Graudal, Simon Tarp, Robin Christensen, Gesche Jürgens

**Affiliations:** 1 Department of Rheumatology IR4242, Copenhagen University Hospital, Rigshospitalet, Denmark; 2 Department of Radiology, Copenhagen University Hospital, Gentofte, Denmark; 3 Musculoskeletal Statistics Unit, the Parker Institute, Department of Rheumatology, Copenhagen University Hospital, Frederiksberg, Denmark; 4 Institute of Sports Science and Clinical Biomechanics, University of Southern Denmark, Odense, Denmark; 5 Department of Clinical Pharmacology, Copenhagen University Hospital, Bispebjerg, Denmark; Universidad Peruana de Ciencias Aplicadas (UPC), Peru

## Abstract

**Background:**

Despite significant cost differences, the comparative effect of combination treatments of disease modifying anti-rheumatic drugs (DMARDs) with and without biologic agents has rarely been examined. Thus we performed a network meta-analysis on the effect of combination therapies on progression of radiographic joint erosions in patients with rheumatoid arthritis (RA).

**Methods and Findings:**

The following combination drug therapies compared versus single DMARD were investigated: Double DMARD: 2 DMARDs (methotrexate, sulfasalazine, leflunomide, injectable gold, cyclosporine, chloroquine, azathioprin, penicillamin) or 1 DMARD plus low dose glucocorticoid (LDGC); triple DMARD: 3 DMARDs or 2 DMARDs plus LDGC; biologic combination: 1 DMARD plus biologic agent (tumor necrosis factor α inhibitor (TNFi) or abatacept or tocilizumab or CD20 inhibitor (CD20i)). Randomized controlled trials were identified in a search of electronic archives of biomedical literature and included in a star-shaped network meta-analysis and reported according to the Preferred Reporting Items for Systematic Reviews and Meta-Analyses (PRISMA) statement protocol. Effects are reported as standardized mean differences (SMD). The effects of data from 39 trials published in the period 1989–2012 were as follows: Double DMARD: −0.32 SMD (CI: −0.42, −0.22); triple DMARD: −0.46 SMD (CI: −0.60, −0.31); 1 DMARD plus TNFi: −0.30 SMD (CI: −0.36, −0.25); 1 DMARD plus abatacept: −0.20 SMD (CI: −0.33, −0.07); 1 DMARD plus tocilizumab: −0.34 SMD (CI: −0.48, −0.20); 1 DMARD plus CD20i: −0.32 SMD (CI: −0.40, −0.24). The indirect comparisons showed similar effects between combination treatments apart from triple DMARD being significantly better than abatacept plus methotrexate (−0.26 SMD (CI: −0.45, −0.07)) and TNFi plus methotrexate (−0.16 SMD (CI: −0.31, −0.01)).

**Conclusion:**

Combination treatment of a biologic agent with 1 DMARD is not superior to 2–3 DMARDs including or excluding LDGC in preventing structural joint damage. Future randomized studies of biologic agents should be compared versus a combination of DMARDs.

## Introduction

In a meta-analysis of 70 randomized controlled trials (RCTs) of rheumatoid arthritis (RA) patients investigating the effect of drug treatment on radiographic joint destruction (erosions), disease modifying anti rheumatic drugs (DMARDs), low-dose glucocorticoids (LDGC), biologic agents, and combinations of these significantly reduced radiographic progression with a relative effect of 48–84% compared with placebo treatment [1]. Although several biologic agents have been investigated as single therapy, biologic treatment is usually given in combination with a DMARD (typically methotrexate) in order to minimize the risk of developing neutralizing antibodies and to improve efficacy. A biologic agent plus methotrexate is superior to single methotrexate and superior to a single biologic agent [1]. Furthermore a combination of DMARDs is superior to a single DMARD [1]. Due to the lack of combination DMARD arms in the studies of biological drugs [1], [2], the comparative effect of combination treatments with and without biologic agents is unclear.

Hitherto only one randomized trial has directly compared the combination of a biologic agent plus methotrexate with a combination of DMARDs [3]. This study and its follow-up study [4] showed no difference between these two treatment principles. Very recently, additionally three studies have confirmed these observations [5]–[7]. Due to the shortage of direct comparisons, network (or mixed treatment comparison (MTC)) meta-analyses [8] have been performed to indirectly compare the effects of different biologic agents [9]–[10]. In contrast, the combination of conventional DMARDs versus biologic agents plus DMARDs have not been analysed in network meta-analyses, although such comparisons seem more interesting due to the cost differences between treatments with and without biologic agents. As our previous study [1] indicated that combination drug treatment was effective irrespective of the drugs involved in the combination, we intended to test the hypothesis that in patients with RA combination treatments of at least two DMARDs, or at least one DMARD plus LDGC or one DMARD plus a biologic agent do not differ significantly in their ability to reduce radiographic joint destruction (erosions) when compared with a single DMARD. Consequently we performed a network meta-analysis of the available direct and indirect evidence from RCTs comparing combination treatment versus single DMARD treatment.

## Methods

The analysis is reported according to the Preferred Reporting Items for Systematic reviews and Meta-Analyses (PRISMA) [11] and supplied with an analysis of consistency between indirect and direct evidence [12]. The first version of a protocol for the present study was performed on October 12, 2010 and was based on our previous meta-analysis [1].

### Definition of network

Unlike a traditional meta-analysis, which summarizes the results of trials that have evaluated the same treatment/placebo combination (direct comparison), a network meta-analysis consist of a network of treatment effects for all possible pairwise comparisons from RCTs, whether or not they have been compared head to head (i.e. include both direct and indirect comparisons). The fundamental principle of the network is that the indirectly compared treatment effects have a common comparator on which they are anchored. In a simple network there is only one common comparator, whereas more complex networks may have several comparators, which are connected in the network.

The disadvantage of complex networks with many anchor treatments is that at least some of the many different treatment principles usually will be unbalanced and thus contribute to heterogeneity, which may complicate the interpretation of the outcome of the analysis. Furthermore, many of the treatments in a complex network often originates from a single study and thus do not benefit from the statistical power, which is the advantage of a conventional meta-analysis. Thus a complex network meta-analysis may result in numerous pairwise comparisons with low power and a high degree of undefined heterogeneity. Consequently, although the universality of the complex models is appealing, it is important to design a network with caution to avoid creating statistical results of limited clinical value.

For instance the total number of treatment principles in our first analysis [1] was 34. If all these principles should be compared in one network meta-analysis the result would be 561 comparisons, many of which would be clinically uninteresting and most of which would have low power. Inclusion of different doses of the same treatment would increase the problem.

In order to minimize the number of low power comparisons and the amount of heterogeneity we intended to create a simple network focussing on the interesting question and eliminating repetition of established evidence on the ability of drugs to reduce inflammation and joint destruction in RA.

First it is established in several conventional meta-analyses of direct comparisons that a single DMARD is better than placebo. Furthermore direct comparisons have shown that DMARDs generally have similar effects. Finally it has been established in direct comparisons that 2–3 DMARDs are better than one DMARD. In addition treatment principles, which are not fully investigated, should be avoided in the network. For instance the 10 known DMARDs can be combined in 45 different double combinations. However only 6 of these combinations have been tested, and therefore it is not possible to determine the most effective of the 45 combinations. Furthermore 4 of the combinations have only been tested in one study. Therefore statistical conclusions based on indirect comparisons of these combinations would be weak. In contrast, a comparison of a group of combination DMARD studies with other treatments would be powerful. The different biologic drugs combined with methotrexate have all been investigated in large studies, and therefore these combinations could all be included in powerful comparisons. Elimination of non-standard doses of biologics, which in direct comparisons have been shown to be inferior, would contribute to the reduction of heterogeneity.

The issue of interest does not only depend on the effect of the treatment, but also on the cost of the treatment. For instance a large difference between cheap DMARDs is interesting, whereas a small difference is not. Similarly a large difference between expensive biologics may be interesting, whereas a small difference is not. In contrast, it would be very interesting if there was only a small or no difference in effect between DMARDs and biologics.

We already know from previous conventional meta-analyses and network meta-analyses that the mutual effects of DMARDs and the mutual effects of biologics are similar, and that biologics as single treatment are better than single DMARD treatment. Furthermore we know the optimal standard dose of the biologics. Considering the 100 fold difference in cost, the remaining interesting question is whether a combination of a standard dose of a biologic plus methotrexate is better than a combination of cheap DMARDs. Consequently it was the intention to create a network to answer that question. Existing evidence was used to simplify the network in order to decrease heterogeneity and increase the power of the comparisons:

1. Placebo controlled single DMARD studies are eliminated, because the effects of single DMARDs are established
2. Single DMARD controlled single DMARD studies are eliminated, because the similar effects of single DMARDs are established
3. The combination DMARD studies are combined in one group and the comparison of different DMARD combinations are eliminated due to lack of investigations and power
4. To ensure the comparability with other network meta-analyses, the different biologic combinations are not combined but compared separately.
5. Only standard doses of biologics are investigated
6. IL1i treatment (anakinra) was excluded as IL1i has been shown to be inferior to other biologics in several network meta-analyses.

### Eligibility criteria

#### Types of studies

Full-length studies published in peer-reviewed journals that were performed according to a RCT design and that scored joint radiographs as the primary or secondary outcome at 2 separate time points with a time interval of at least 3 months were included, irrespective of sample size and publication year.

#### Types of participants

Patients with RA diagnosed according to the 1958 or the 1987 criteria of the American College of Rheumatology (ACR; formerly, the American Rheumatism Association) were included. In studies performed before 1959, the stated study definitions of RA were accepted.

#### Type of outcome

The outcome was the difference between follow-up radiographic erosion score and baseline radiographic erosion score.

#### Types of intervention

As our previous meta-analysis [1] showed no statistically significant difference in radiographic progression between methotrexate (Mt), sulfasalazine (Su), cyclosporine (Cs), leflunomide (Lf) and injectable gold (Au, ij), we included combination DMARD studies, which had one of these effective DMARDs in the single DMARD arm, but excluded those that included the less effective DMARDs (chloroquine (Cl), D-penicillamine (Dp) and Dp analogue bucillamin (Bu), azathioprine (Az), cyclophosphamide (Cph) and peroral gold (Au, po)) in the single DMARD arm. Furthermore, we showed that LDGC, defined as maximally 7.5 mg prednisone or prednisolone per day, had an effect similar to the effective DMARDs [1], and therefore LDGC was included as a DMARD equivalent. Any DMARD was allowed in the combination arm. Finally, we included combination treatments of methotrexate plus TNF inhibitors (etanercept (Et), infliximab (In), adalimumab (Ad), certolizumab (Cz), and golimumab (Go)), methotrexate plus abatacept (Ab), methotrexate plus tocilizumab (Tz), and methotrexate plus CD20 inhibitors (rituximab (Rt), ocrelizumab (Oc)).

### Information sources

Trials were identified by searching the electronic databases (PubMed, the Cochrane database, and ClinicalTrials.gov) and by scanning the lists of references from the identified randomized trials.

### Search methods for identification of studies

The search was based on the following combination of search terms:

“rheumatoid arthritis and randomized and methotrexate OR rheumatoid arthritis and randomized and sulfasalazine OR rheumatoid arthritis and randomized and leflunomide OR rheumatoid arthritis and randomized and gold OR rheumatoid arthritis and randomized and cyclosporine OR rheumatoid arthritis and randomized and infliximab OR rheumatoid arthritis and randomized and etanercept OR rheumatoid arthritis and randomized and adalimumab OR rheumatoid arthritis and randomized and certolizumab OR rheumatoid arthritis and randomized and golimumab OR rheumatoid arthritis and randomized and tocilizumab OR rheumatoid arthritis and randomized and abatacept OR rheumatoid arthritis and randomized and rituximab OR rheumatoid arthritis and randomized and ocrelizumab OR rheumatoid arthritis and randomized and ofatumumab OR rheumatoid arthritis and randomized and glucocorticoid OR rheumatoid arthritis and randomised and methotrexate OR rheumatoid arthritis and randomised and sulfasalazine OR rheumatoid arthritis and randomised and leflunomide OR rheumatoid arthritis and randomised and gold OR rheumatoid arthritis and randomised and cyclosporine OR rheumatoid arthritis and randomised and infliximab OR rheumatoid arthritis and randomised and etanercept OR rheumatoid arthritis and randomised and adalimumab OR rheumatoid arthritis and randomised and certolizumab OR rheumatoid arthritis and randomised and golimumab OR rheumatoid arthritis and randomised and tocilizumab OR rheumatoid arthritis and randomised and abatacept OR rheumatoid arthritis and randomised and rituximab OR rheumatoid arthritis and randomised and ocrelizumab OR rheumatoid arthritis and randomised and ofatumumab OR rheumatoid arthritis and randomised and glucocorticoid.”

### Data collection

#### Selection of trials

Titles were screened, abstracts read, and possible papers retrieved. Trials fulfilling eligibility criteria were included in the systematic review.

#### Data extraction

Eligibility assessment, data collection and risk of bias assessment were performed independently by two authors and disagreement resolved by consensus. All data were entered into standardized extraction forms.

#### Data items

Mean radiographic scores and standard deviations (SD) were assessed based on the change scores from baseline to follow-up for each treatment arm. In addition the following variables were recorded: Study identification, year of publication, scoring system, initial radiographic score, maximum radiographic score of scoring system, number of patients in each treatment arm, duration of RA at baseline, duration of study, DMARD inadequate response (i.e. whether included patients previously had had an inadequate response to a least one DMARD), strategy change (i.e. whether a change of treatment strategy was allowed during the course of the study) and mean daily glucocorticoid use in all treatment arms. We used the baseline radiographic score, the maximum radiographic score of scoring system and the duration of RA to calculate the percentage annual radiographic progression rate (PARPR) [1] in the period before baseline as a marker of disease activity at baseline.

### Risk of bias in individual studies

Six different risk-of-bias domains defined by Cochrane [13] were assessed on the outcome level: sequence generation, allocation concealment, study blinding, outcome assessor blinding, incomplete outcome data and selective outcome reporting. In addition we included radiographic sequence blinding and company sponsoring as risk of bias domains.

### Measures of treatment effect

For each randomized combination drug group and single DMARD group the difference between follow-up radiographic erosion score and baseline radiographic erosion score and the corresponding SDs were recorded. The difference between the mean effect in the combination drug group and the single DMARD group was the treatment effect.

### Data analysis

#### Unit of analysis issues

If radiographic scoring was performed more than once during follow-up, the scoring with the most complete data was recorded. In trials with multi dose arms, only the defined standard dose arm was included. If the treatment arms of multi-armed trials consisted of different combination treatments (direct comparisons), these treatment arms were included in the network meta-analysis and additionally analyzed separately for the purposes of a consistency analysis of indirect comparisons versus direct comparisons. In this case the shared control group was split into a number of subgroups corresponding to the number of treatment arms to avoid multiple count of the control group.

#### Missing data

In articles where the median, but not the mean, was given, the median value was used in the calculations. If SD was not given, it could often be calculated from a 95% confidence interval, a standard error or a p-value [13]. An interquartile range (2 quartiles) was made equivalent to 1.35 x SD [13] and a full range was converted to an SD according to a conversion factor defined by Walther and Yao [14].

#### Heterogeneity

Heterogeneity between studies was tested statistically for all studies and each intervention by a χ^2^ (chi square) test, and quantified by means of the I^2^ statistic, which describes the percentage of the variability in effect estimates that is due to heterogeneity rather than sampling error: I^2^, 0%–30%, unimportant heterogeneity; I^2^, 30%–60%, moderate heterogeneity; I^2^, >60%, substantial heterogeneity [13]. Both fixed and random effect models were used, but the existence of statistically significant heterogeneity would determine whether a fixed effect model (non-significant heterogeneity test) or a random effect model (significant heterogeneity test) would be used in the primary or secondary analysis [13]. Potential heterogeneity would be explored by means of subgroup analyses of extracted data.

#### Outcome data synthesis

A network meta-analysis consist of a network of treatment effects for all possible pairwise comparisons from RCTs, whether or not they have been compared head to head (i.e. include both direct and indirect comparisons) [7], [8]. We used a stepwise approach [15], [16], first performing multiple pairwise meta-analyses of the direct comparisons of each of the combination treatments versus single DMARD followed by an indirect comparison of the pooled results of each of these meta-analyses. As the outcome measure (radiographic progression) was estimated at different time points (6–24 months) and as the maximum score of the different scoring systems (Sharp, Larsen) differed, we standardized the outcome measure by dividing the outcome with the SD, thus converting the outcome unit to the unitless standardized mean difference (SMD) [13]. Consequently, we interpreted our analyses of the pairwise meta-analyses on the basis of the SMD, whereas the indirect comparisons were performed as weighted mean differences of the SMDs calculated in the pairwise meta-analyses.

#### Consistency analysis

Consistency analyses of the effects obtained in the trials directly comparing combination treatments versus the effects obtained by means of the exclusively indirect comparisons were performed to explore possible differences between the direct and the indirect comparisons [12].

#### Risk of bias across studies

Each of the above eight assessed risk of bias domains were evaluated in 3 groups: A: Low risk; B: Unclear risk; C: High risk [13]. In addition publication bias was evaluated visually by means of a funnel plot in which the effect of each trial was plotted by the inverse of its standard error [13].

#### Additional analyses

The outcome effect (radiographic progression) of combination DMARD treatments including LDGC was compared versus combination DMARD treatments not including LDGC.

Measures of bias domains and of other possible confounders were compared between the combination treatment groups with the purpose of performing sensitivity analyses for those, which differed. The outcome effect was compared between the grading (A, B, C) of the relevant bias domains and between the upper and lower 50% percentiles of possible confounders of continuous variables (PARPR (as a marker of disease activity at baseline), disease duration, differences in the mean use of glucocorticoids) and between groups of possible confounders of category variables (DMARD inadequate response and strategy change).

#### Data synthesis method

The combined effect measures of the direct comparisons of the individual combination treatments, the indirect comparisons of the combined effect measures of the individual combination treatments, the consistency analyses and the additional analyses were compared by means of the inverse variance method in Review Manager (RevMan) (Computer program), version 5.1. Copenhagen: The Nordic Cochrane Centre, the Cochrane Collaboration, 2008 [13].

## Results

### Trial selection

**Figure 1:**
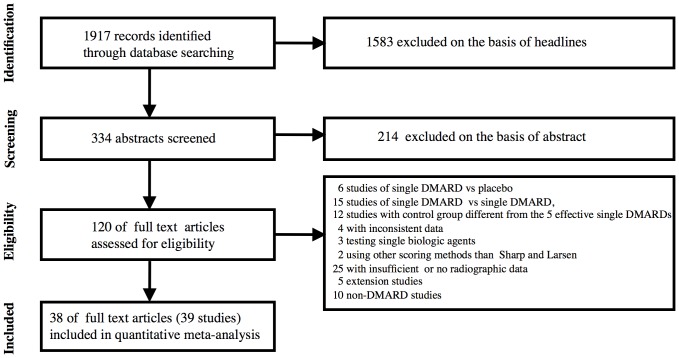
Flow diagram of literature search.

The search was repeated during the review period, by two authors by turns. The final search was performed July 5, 2012. A flow diagram of the literature search is shown in Figure 1.

The PubMed search revealed 1917 references. A search of ClinicalTrials.gov using the key-words “rheumatoid arthritis” and “radiographic progression” revealed 3 published studies with radiographic data, which also were identified during our primary search, 1 published study with no radiographic data and 2 finished but not published studies out of a total of 21 ongoing studies.

This search was supplied with a search in Cochrane Central Register of Controlled Trials using the terms “rheumatoid arthritis and radiographic progression” or “rheumatoid arthritis and joint destruction” resulting in 65 hits, none of which supplied the list of included studies.

After eliminating references which were considered irrelevant according to the headlines, 334 abstracts were read. On the basis of the abstracts 120 articles were retrieved in full length. From these a total of 38 references were identified (Figure 1). Until December 31 2009 the present search identified all 28 combination studies [3], [17]–[43] identified in our previous search [1] plus one additional study published in 2005 [44]. In addition the present search revealed three new references [45]–[47] (4 investigations) published in 2011 and 6 studies published in 2012 [48]–[53]. In total 38 “combination treatment” references (39 trials, 45 treatment groups) were included.

### Characteristics of included studies

**Figure 2:**
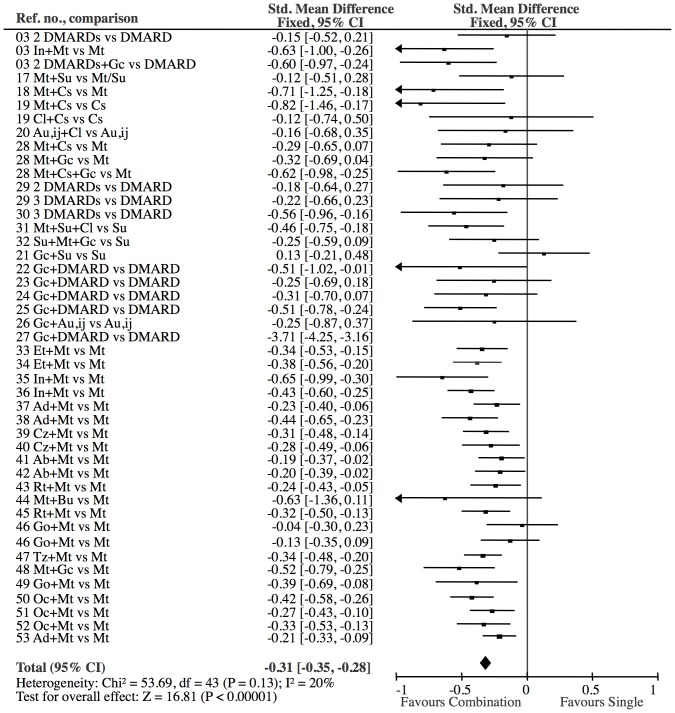
Combination treatment versus single DMARD. The effect on all studies is −0.33 SMD (CI: −0.36, −0.29). Test for overall effect: Z = 17.66 (P<0.00001). Heterogeneity: Chi^2^ = 201.54, df = 44 (P<0.00001); I^2^ = 78%. One study [27] contributed to heterogeneity due an extreme effect (−3.71 SMD). The elimination of this study resulted in a little more conservative estimate (−0.31 SMD (CI:−0.35, −0.28), Z = 16.81), but eliminated the significant heterogeneity (I^2^ = 20, p = 0.13). Consequently, reference [27] was excluded from all comparisons. N, combination: 6725; N, single: 5446.

All were parallel studies. Individual study characteristics and risk of bias domains are shown in Table 1. A forest plot of the individual study results is shown in Figure 2. Heterogeneity seemed to be unimportant (I^2^ = 20%, p = 0.13).

**Table 1:** Study Characteristics and Risk of Bias Factors.

Reference no.	Sequence generation	Concealed allocation	Study blinding	Outcome blinding	Radiographic sequence	Incomplete outcome	Sponsor	Test drug	Treatment group	N_(radiograph)	Duration RA, years	Scoring system	Initial radiographic score	Radiographic score, Max	Duration of study, months	PARPR	Mean Dose GC mg	Strategy change allowed	DMARD inadequate response
[3]	A	A	C	A	A	C	C	Mt	Single	115	0,5	Sharp	2,0	280	12	1,4	0,03	Yes	No
[3]	A	A	C	A	A	C	C	MtSu	Double	110	0,5	Sharp	2,0	280	12	1,4	0,04	Yes	No
[3]	A	A	C	A	A	C	C	MtSuGc	Triple	125	0,5	Sharp	2,0	280	12	1,4	0,01	Yes	No
[3]	A	A	C	A	A	C	C	InMt	TNFiMt	119	0,5	Sharp	2,0	280	12	1,4	0,02	Yes	No
[17]	B	B	A	A	C	C	C	Mt	Single	49	1,5	Sharp	3,8	280	12	0,9	0,00	No	No
[17]	B	B	A	A	C	C	C	Su	Single	46	0,9	Sharp	2,8	280	12	1,1	0,00	No	No
[17]	B	B	A	A	C	C	C	MtSu	Double	49	0,9	Sharp	4,3	280	12	1,7	0,00	No	No
[18]	B	B	C	A	C	C	A	Mt	Single	30	0,9	Sharp	1,2	280	12	0,5	2,10	Yes	No
[18]	B	B	C	A	C	C	A	MtCs	Double	28	0,9	Sharp	1,1	280	12	0,4	2,10	Yes	No
[19]	B	B	C	A	B	C	B	Cs	Single	30	1,3	Larsen	14,5	200	12	5,6	2,20	No	No
[19]	B	B	C	A	B	C	B	CsMt	Double	30	1,3	Larsen	13,5	200	12	5,2	3,25	No	No
[19]	B	B	C	A	B	C	B	CsCl	Double	29	1,5	Larsen	13,7	200	12	4,6	2,15	No	No
[20]	B	B	A	A	A	C	C	Au	Single	32	2,0	Larsen	30,5	240	12	6,4	0,00	No	No
[20]	B	B	A	A	A	C	C	AuCl	Double	27	2,0	Larsen	31,2	240	12	6,5	0,00	No	No
[21]	A	A	A	A	C	C	A	Su	Single	66	1,0	Sharp	6,0	448	12	1,3	0,09	No	No
[21]	A	A	A	A	C	C	A	SuGc	Double	64	1,0	Sharp	8,0	448	12	1,8	0,09	No	No
[22]	B	B	A	B	B	C	A	Mt	Single	30	16,0	Larsen	37,0	240	12	1,0	0,09	Yes	No
[22]	B	B	A	B	B	C	A	MtGc	Double	32	13,0	Larsen	47,0	240	12	1,5	0,06	Yes	No
[23]	A	B	C	A	A	C	A	Mt	Single	39	8,5	Larsen	31,5	160	12	2,3	0,04	Yes	No
[23]	A	B	C	A	A	C	A	MtGc	Double	43	2,8	Larsen	28,5	160	12	6,4	0,05	Yes	No
[24]	B	A	A	A	A	C	A	Su	Single	49	1,3	Larsen	6,2	140	12	3,4	0,00	No	No
[24]	B	A	A	A	A	C	A	SuGc	Double	54	1,3	Larsen	2,7	140	12	1,5	0,00	No	No
[25]	A	B	C	A	C	C	A	Mt	Single	115	0,5	Sharp	1,9	280	12	1,4	0,62	Yes	No
[25]	A	B	C	A	C	C	A	MtGc	Double	99	0,5	Sharp	1,9	280	12	1,4	0,28	Yes	No
[26]	B	B	A	B	B	A	A	Au	Single	20	2,5	Sharp	17,0	280	10	2,4	0,04	Yes	No
[26]	B	B	A	B	B	A	A	AuGc	Double	20	1,8	Sharp	11,0	280	10	2,2	0,01	Yes	No
[27]	A	B	A	A	C	C	C	Mt	Single	72	0,8	Sharp	4,7	280	12	2,1	0,00	No	No
[27]	A	B	A	A	C	C	C	MtGc	Double	70	0,8	Sharp	6,0	280	12	2,7	0,00	No	No
[28]	A	A	A	A	B	A	A	Mt	Single	117	0,2	Larsen	7,0	240	24	14,6	0,82	Yes	No
[28]	A	A	A	A	B	A	A	MtCs	Double	119	0,3	Larsen	8,0	240	24	11,1	0,82	Yes	No
[28]	A	A	A	A	B	A	A	MtGc	Double	115	0,4	Larsen	5,0	240	24	5,2	0,82	Yes	No
[28]	A	A	A	A	B	A	A	MtCsGc	Triple	116	0,3	Larsen	5,0	240	24	6,9	0,82	Yes	No
[29]	B	B	C	A	B	C	B	Mt	Single	57	2,3	Larsen	32,8	200	24	7,1	1,50	Yes	No
[29]	B	B	C	A	B	C	B	MtSu	Double	56	2,5	Larsen	33,6	200	24	6,7	1,50	Yes	No
[29]	B	B	C	A	B	C	B	MtSuCl	Triple	58	2,2	Larsen	33,4	200	24	7,6	1,50	Yes	No
[30]	A	A	C	A	C	C	A	Su	Single	47	1,7	Sharp	32,0	280	18	6,7	1,15	Yes	No
[30]	A	A	C	A	C	C	A	MtSuCl	Triple	52	1,6	Sharp	28,0	280	18	6,3	4,08	Yes	No
[31]	A	A	C	A	B	A	A	Mt	Single	98	0,7	Larsen	2,0	240	24	1,2	3,59	No	No
[31]	A	A	C	A	B	A	A	MtSuCl	Triple	97	0,6	Larsen	2,0	240	24	1,4	5,14	No	No
[32]	A	A	A	A	C	C	B	Su	Single	65	0,3	Sharp	5,0	280	13	6,0	0,27	No	No
[32]	A	A	A	A	C	C	B	SuMtGc	Triple	70	0,3	Sharp	2,0	280	13	2,4	0,27	No	No
[33]	B	A	A	A	A	C	C	Mt	Single	212	6,8	Sharp	11,5	280	12	0,6	3,20	No	No
[33]	B	A	A	A	A	C	C	EtMt	TNFiMt	218	6,8	Sharp	9,5	280	12	0,5	3,10	No	No
[34]	A	A	A	A	A	C	C	Mt	Single	230	0,8	Sharp	5,0	448	12	1,4	2,50	No	No
[34]	A	A	A	A	A	C	C	EtMt	TNFiMt	246	0,7	Sharp	5,0	448	12	1,6	2,45	No	No
[35]	B	B	B	A	A	C	C	Mt	Single	64	11,0	Sharp	82,0	280	12	2,7	3,20	No	Yes
[35]	B	B	B	A	A	C	C	InMt	TNFiMt	71	10,0	Sharp	79,0	280	12	2,8	3,15	No	Yes
[36]	A	A	A	A	A	C	C	Mt	Single	226	0,9	Sharp	8,3	280	12	3,3	1,90	Yes	No
[36]	A	A	A	A	A	C	C	InMt	TNFiMt	306	0,8	Sharp	8,8	280	12	3,9	1,85	Yes	No
[37]	B	B	A	A	A	A	C	Mt	Single	251	0,8	Sharp	13,6	230	12	7,4	2,27	No	No
[37]	B	B	A	A	A	A	C	AdMt	TNFiMt	267	0,7	Sharp	11,0	230	12	6,8	2,40	No	No
[38]	B	B	A	A	A	C	C	Mt	Single	172	10,9	Sharp	37,2	230	12	1,5	2,71	Yes	Yes
[38]	B	B	A	A	A	C	C	AdMt	TNFiMt	183	11,0	Sharp	41,4	230	12	1,6	2,46	Yes	Yes
[39]	B	B	A	A	A	A	C	Mt	Single	199	6,2	Sharp	20,0	440	12	0,7	1,70	No	Yes
[39]	B	B	A	A	A	A	C	CzMt	TNFiMt	393	6,1	Sharp	20,0	440	12	0,7	1,70	No	Yes
[40]	B	B	A	A	A	A	C	Mt	Single	127	5,6	Sharp	23,1	280	6	1,5	2,85	No	Yes
[40]	B	B	A	A	A	A	C	CzMt	TNFiMt	246	6,1	Sharp	19,0	280	6	1,1	2,75	No	Yes
[41]	A	A	B	A	A	A	C	Mt	Single	195	8,9	Sharp	21,8	145	12	1,7	3,45	Yes	Yes
[41]	A	A	B	A	A	A	C	AbMt	ABAMt	391	8,5	Sharp	21,7	145	12	1,8	3,60	Yes	Yes
[42]	B	B	A	A	B	C	C	Mt	Single	223	0,5	Sharp	4,8	145	12	6,6	4,56	Yes	No
[42]	B	B	A	A	B	C	C	AbMt	ABAMt	218	0,5	Sharp	5,4	145	12	7,4	3,63	Yes	No
[43]	B	B	C	A	A	C	C	Mt	Single	184	11,6	Sharp	46,2	145	12	2,7	3,05	No	Yes
[43]	B	B	C	A	A	C	C	RtMt	CD20iMt	273	12,0	Sharp	46,2	145	12	2,7	3,25	No	Yes
[44]	A	A	A	A	B	C	A	Mt	Single	15	0,7	Sharp	24	448	22	7,7	1.6	Yes	No
[44]	A	A	A	A	B	C	A	MtBu	Double	15	0,8	Sharp	14	448	22	3,9	0.8	Yes	No
[45]	A	A	A	A	A	A	C	Mt	Single	232	0,90	Sharp	3,70	145	12	2,8	2,40	No	No
[45]	A	A	A	A	A	A	C	RtMt	CD20iMt	244	0,90	Sharp	3,50	145	12	2,7	2,20	No	No
[46a]	B	A	A	B	B	A	C	Mt	Single	160	2,9	Sharp	19,7	448	12	1,5	3,40	Yes	No
[46a]	B	A	A	B	B	A	C	GoMt	TNFiMt	159	3,5	Sharp	18,7	448	12	1,2	3,50	Yes	No
[46b]	B	A	A	B	B	A	C	Mt	Single	133	6,5	Sharp	36,7	448	12	1,3	4,80	Yes	Yes
[46b]	B	A	A	B	B	A	C	GoMt	TNFiMt	89	4,5	Sharp	29,7	448	12	1,5	5,60	Yes	Yes
[47]	B	B	A	B	B	B	C	Mt	Single	393	9	Sharp	28,8	145	12	2,2	3,45	Yes	Yes
[47]	B	B	A	B	B	B	C	TzMt	TZMt	398	9,3	Sharp	29,1	145	12	2,2	3,10	Yes	Yes
[48]	A	A	A	A	C	C	A	Mt	Single	110	0,6	Sharp	0	280	24	0,0	0	Yes	No
[48]	A	A	A	A	C	C	A	MtGc	Double	112	0,6	Sharp	0	280	24	0,0	0	Yes	No
[49]	B	B	A	A	A	B	C	Mt	Single	84	8,7	Sharp	30,8	280	6	1,3	NA	Yes	Yes
[49]	B	B	A	A	A	B	C	GoMt	TNFiMt	81	8,8	Sharp	32,1	280	6	1,3	NA	Yes	Yes
[50]	B	A	A	A	B	C	C	Mt	Single	285	7,2	Sharp	NA	448	11	NA	3,54	No	Yes
[50]	B	A	A	A	B	C	C	OcMt	CD20iMt	324	7,6	Sharp	NA	448	11	NA	3,83	No	Yes
[51]	B	B	A	A	C	C	C	Mt	Single	282	11,8	Sharp	NA	448	11	NA	4,07	No	Yes
[51]	B	B	A	A	A	C	C	OcMt	CD20iMt	277	12,3	Sharp	NA	448	11	NA	3,81	No	Yes
[52]	B	A	A	A	B	C	C	Mt	Single	196	1,23	Sharp	13,3	448	12	2,4	2	No	No
[52]	B	A	A	A	B	C	C	OcMt	CD20iMt	194	1,2	Sharp	12,4	448	12	2,3	2,11	No	No
[53]	B	A	A	A	B	A	C	Mt	Single	514	4,5	Sharp	5,1	280	6	0,4	2,3	No	No
[53]	B	A	A	A	B	A	C	AdMt	TNFiMt	508	4	Sharp	5,4	280	6	0,5	2,05	No	No

*Percentage of Annual Radiographic Progression Rate

### Description of network

On the basis of the included treatment arms and doses, we defined 6 combination treatments versus single DMARD:

1. Two DMARDs/LDGC (Double);
2. Three DMARDs/LDGC (Triple);
3. Standard dose of TNFi (Infliximab: 3 mg/kg/8 weeks; etanercept: 50 mg/1 week; adalimumab: 40 mg/2 weeks; certolizumab: 200 mg/2 weeks; golimumab: 50 mg/4 weeks);
4. Standard dose of CD20 inhibitor treatment (rituximab 2 g/6 months; ocrelizumab 1 g/6 months);
5. Abatacept 10 mg/kg/4 weeks;
6. Tocilizumab 8 mg/kg/4 weeks.

**Figure 3:**
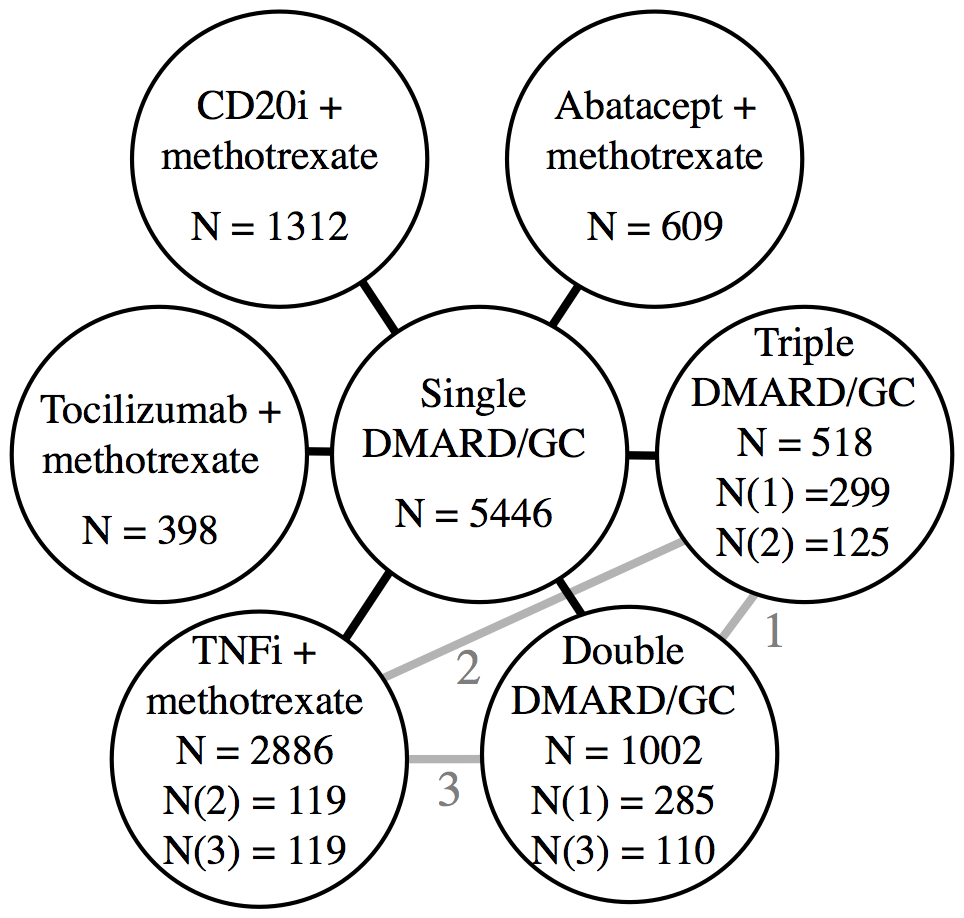
Star shaped network showing the 6 different combination treatments anchored on single treatment as the common comparator. The loops (grey lines) with corresponding numbers (1, 2, 3) show the subgroups, which were directly compared in addition to being indirectly compared. N indicates the number of patients in the groups.

The star shaped network is shown in Figure 3. As one study included a direct comparison between TNFi, double and triple [3] and additionally two studies included direct comparisons between double and triple [28], [29], the star includes loops to indicate the direct comparisons between TNFi, double and triple.

### Synthesis of results

**Figure 4:**
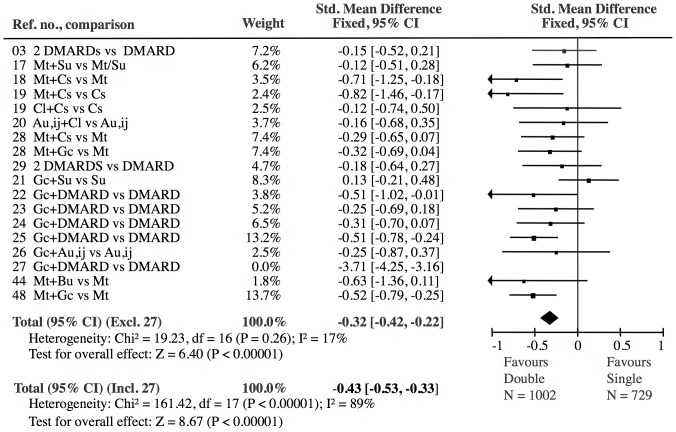
Double DMARD versus single DMARD: The effect of the Double DMARD treatment was highly significant (Z = 6.40). All 18 Double studies showed heterogeneity (I^2^ = 89%). The exclusion of one reference [27], which had an extreme effect (−3.71 SMD), eliminated the significant heterogeneity (I^2^ = 17%).

**Figure 5:**
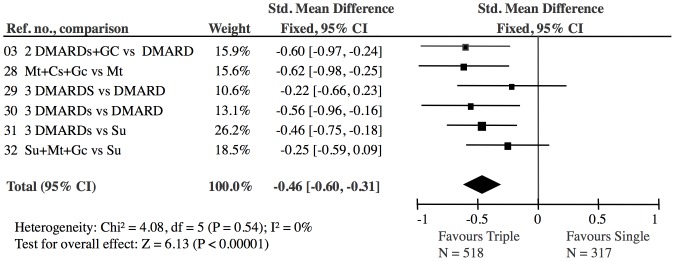
Triple DMARD versus single DMARD: The effect of the Triple DMARD treatment was highly significant (Z = 6.13). The 6 Triple studies showed no heterogeneity (I^2^ = 0).

**Figure 6:**
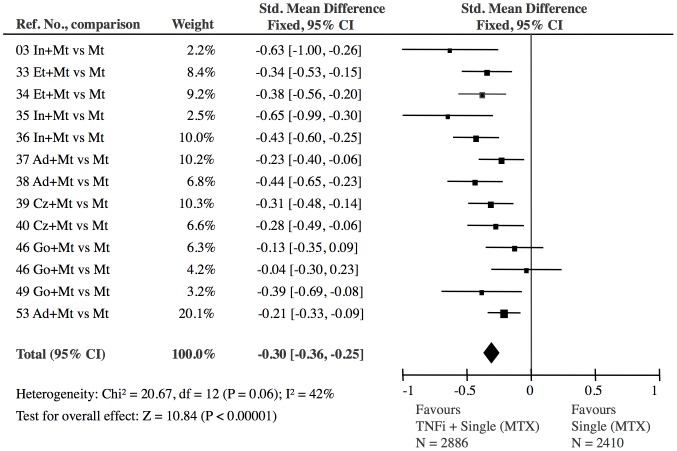
TNF inhibitor combined with methotrexate versus single DMARD (methotrexate): The effect of TNF inhibitor was highly significant (Z = 10.84). The 13 TNF inhibitor studies showed no significant heterogeneity (I^2^ = 42%, p = 0.06). The borderline heterogeneity was due to two golimumab studies (GoBefore, GoForward) [46]. The exclusion of these, did, however, not change the overall result (−0.33 SMD (CI: −0.39, −0.27)).

**Figure 7:**
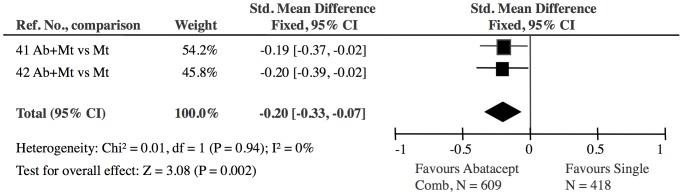
Abatacept combined with methotrexate versus single DMARD (methotrexate): The effect of abatacept was significant (Z = 3.08). The 2 abatacept studies showed no heterogeneity (I^2^ = 0).

**Figure 8:**
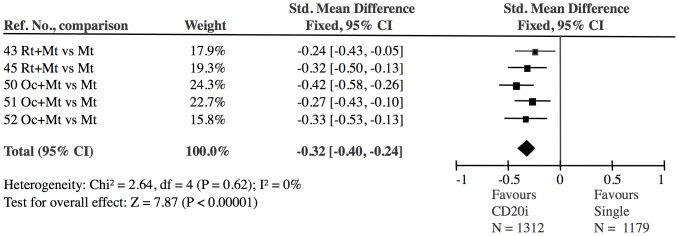
CD20 inhibitor treatment combined with single DMARD versus single DMARD: The effect of CD20 inhibitor treatment was highly significant (Z = 7.87). The 5 CD20 inhibitor studies showed no heterogeneity (I^2^ = 0).

**Figure 9:**
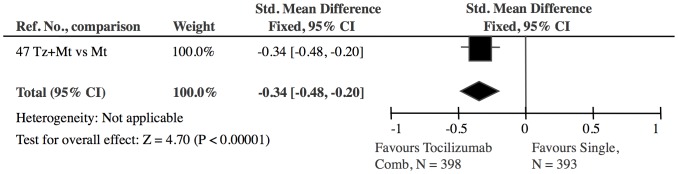
Tocilizumab combined with methotrexate versus single DMARD (methotrexate): The effect of tocilizumab is significant (Z = 4.70).

Only one study [27] contributed to heterogeneity in the analyses of all 45 treatment groups (I^2^ = 78%) (Figure 2) and in the analysis of double DMARD vs. single DMARD (I^2^ = 89%) (Figure 4). All other heterogeneity analyses were non-significant (I^2^ varying in the range 0–42%, Figures 5–9). Consequently we eliminated this study [27] from the statistical analyses (reducing I^2^ to 17–20%) and used a fixed effect model in the primary analyses and a random effect model in the secondary analyses. The results of the conventional meta-analyses of the 6 combination treatments are shown in Figures 4–9. The borderline heterogeneity in the TNFi analysis (I^2^ = 42%) (Figure 6) was due to two golimumab studies [46]. Elimination of these studies reduced heterogeneity (I^2^ = 27%) but did not change the overall result (SMD: −0.33 (CI: −0.39, −0.27)).

**Figure 10:**
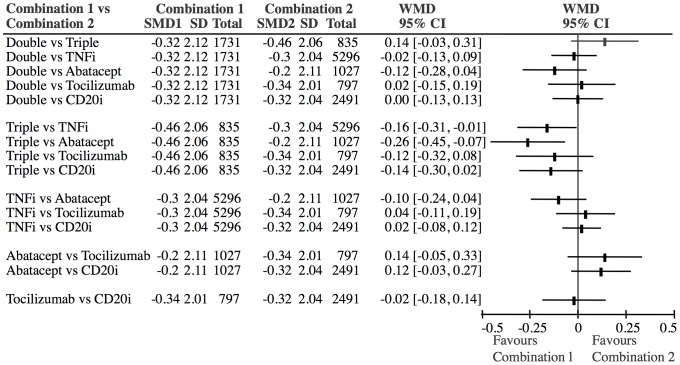
Indirect comparisons of different combination treatments. There is a trend towards triple treatment being superior to abatacept and TNFi. All other differences between the combination treatments are non-significant. Abbreviations: SMD: Standardized mean difference. WMD: Weighted mean difference (SMD1-SMD2).

Because all interventions are connected in the network (i.e. each pair has a path from one to the other) indirect comparisons can be performed for each of the combination treatments in the star versus each other. Figure 10 shows the results of the indirect comparisons of the 6 combination treatments. The effects varied between −0.46 SMD (triple) and −0.20 SMD (abatacept). Statistically, triple treatment with DMARDs was a little better than abatacept plus methotrexate (−0.26 SMD (CI: −0.45, −0.07)) and TNFi plus methotrexate (−0.16 SMD (CI: −0.31, −0.01)), but no other significant differences between the different combination treatments were identified (Figure 10).

### Risk of bias across studies

The cumulated grade (A, B, C) frequencies are shown in Table 2. Six of the eight bias domains are predominantly graded as being of low (A) or unclear (B) risk, whereas two domains (incomplete outcome reporting and study sponsoring) are predominantly classified as being of high risk. Concerning the 6 Cochrane bias domains, 28 of 39 trials contained at least one high risk (C) grade.

**Table 2:** Observed Frequencies of bias factors for treatment groups.

	Double	Triple	TNFi	ABA	CD20i	TZ	?^2^	p
Sequence generation								
A	9	5	3	1	1	0		
B	9	1	10	1	4	1		
C	0	0	0	0	0	0	8.3	0.14
Allocation concealment								
A	7	5	7	1	3	0		
B	11	5	6	1	2	1		
C	0	0	0	0	0	0	4.8	0.44
Study blinding								
A	11	2	11	1	4	1		
B	0	0	1	1	0	0		
C	7	4	1	0	1	0	19.7	0.03
Outcome blinding								
A	16	11	6	2	5	0		
B	2	2	1	0	0	1		
C	0	0	0	0	0	0	9.7	0.09
Radiographic sequence								
A	4	1	10	1	3	0		
B	8	3	3	1	2	1		
C	6	2	0	0	0	0	16.3	0.09
Incomplete outcome data								
A	3	2	6	1	1	0		
B	0	0	1	0	0	1		
C	15	4	6	1	4	0	27.7	0.002
Selective outcome reporting								
A	18	6	13	2	5	1		
B	0	0	0	0	0	0		
C	0	0	0	0	0	0	-	-
Sponsorship								
A	11	3	0	0	0	0		
B	3	2	0	0	0	0		
C	4	1	13	2	5	1	30.1	0.001

**Figure 11:**
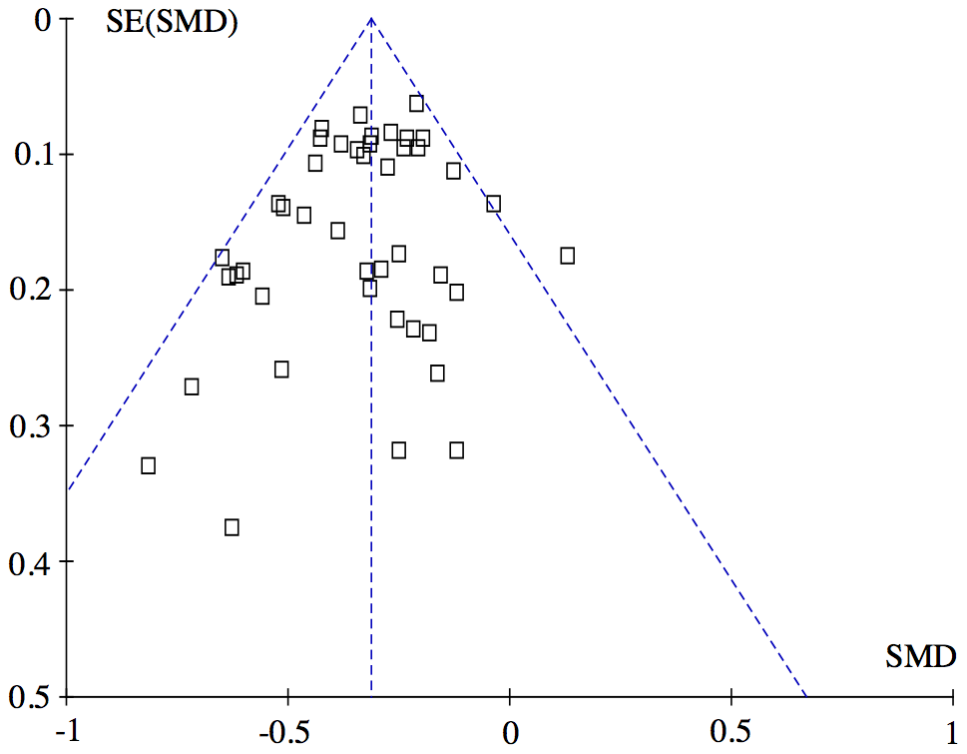
Funnel plot of all combination studies ([27] eliminated). The left lower corner is empty compared with the right lower corner. This asymmetry may indicate that small studies with no effect was not published (publication bias). However, this asymmetry is quantitatively small, and probably does not affect the overall result. Exclusion of the three lower right studies [18], [19], [44] to eliminate the asymmetry did not change the overall result shown in Figure 2: −0.31 SMD (CI: −0.35, −0.27), test for overall effect: Z = 16.49 (P<0.00001). Heterogeneity: Chi^2^ = 48.41, df = 40 (P = 0.17); I^2^ = 17%. Abbreviations: SMD: Standardized mean difference.

A funnel plot indicates a minor degree of publication bias (Figure 11).

### Consistency analysis

Three trials [3], [28], [29] of the 39 trials contributed with treatment arms to three combination treatment groups (TNFi, Double and Triple). Pairwise consistency analyses of the SMD effects obtained in the trials directly comparing combination treatments versus the SMD effects obtained by means of the exclusively indirect comparisons were performed to explore possible differences between the direct and the indirect comparisons.

Triple versus Double: Direct comparison (n = 584) versus indirect comparison (n = 1616): Weighted mean difference  = 0.20 SMD (CI: −0.08, 0.48).

Double versus TNFi plus methotrexate:

1. Direct comparison (BeSt study [3], 1. year data) (n = 229) versus indirect comparison (n = 6722): Weighted mean difference  = 0.55 SMD (CI: 0.28, 0.82).
2. Supplementary analysis including the second year data from the BeSt study [4]: Direct comparison (n = 236) versus indirect comparison (n = 6722): Weighted mean difference  = 0.05 SMD (CI: −0.32, 0.42).

Triple versus TNFi plus methotrexate: Direct comparison (n = 244) versus indirect comparison (n = 5810): Weighted mean difference  = 0.23 SMD (CI: −0.07, 0.53).

### Additional analyses

Using a random effect model instead of a fixed effect model eliminated the small significant difference between triple DMARD and TNFi (weighted mean difference: −0.14 SMD (CI: −0.30; 0.02)), but all other indirect comparisons as shown in Figure 10 were unchanged.

**Figure 12:**
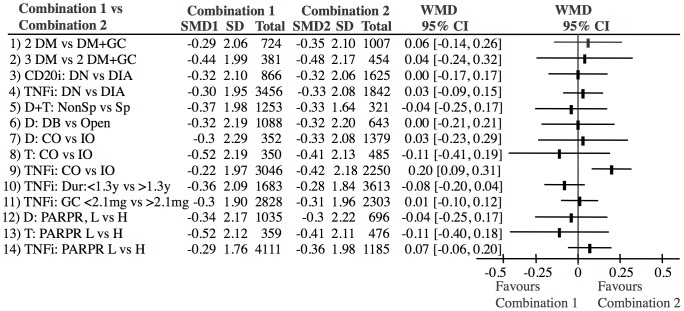
Analyses of bias factors and confounders, which differed significantly across treatment groups. Only 1 bias factor (TNFi studies: Complete outcome versus incomplete outcome, line 9) had a significant influence on the outcome. Abbreviations: SMD: Standardized mean difference. WMD: Weighted mean difference (SMD1-SMD2); DM: DMARD; GC: Glucocorticoid; DN: DMARD naive; DIA: DMARD inadequate responder; D: double; T: Triple; Sp: Sponsoring; DB: double-blind; CO: Complete outcome; IO: Incomplete outcome; Dur: Disease duration at baseline; PARPR: Percentage of annual radiographic progression rate; L: low; H: High.

There was no difference between DMARD combination studies using LDGC as a DMARD equivalent and those using only DMARDs (Figure 12, lines 1–2). There was no difference between biologic studies performed in DMARD naïve (DN) patients and DMARD inadequate responders (DIA) (Figure 12, lines 3–4).

Table 3 shows other possible confounders across treatment groups. Sensitivity analyses were performed for the bias domains (Table 2) and possible confounding variables (Table 3), which differed across studies and the results are shown in Figure 12. The results of these analyses showed that these factors did not influence the results significantly (Figure 12, lines 5–14) with the exception TNFi studies with incomplete outcome reporting (high risk of bias), which had a significantly higher effect than those with complete outcome reporting (low risk of bias) (Figure 12, line 9).

**Table 3:** Other possible confounders across treatment groups.

	Double	Triple	TNFi	ABA	CD20i	TZ	p
N	18	6	13	2	5	1	
Duration (years) of RA at baseline							
Mean	2.1	0.9	5.0	4.6	6.7	9.2	
SD	3.4	0.8	3.6	4.7	5.1	0.2	0.0001
Percentage of annual radiographic progression rate at baseline							
Mean	3.5	5.2	1.9	4.4	2.6	2.2	
SD	3.3	3.9	1.7	3.1	0.2	0.04	0.03
Glucocorticoid use during study							
Mean	0.6	1.6	2.6	3.8	3.0	3.3	
SD	0.9	1.7	1.2	0.5	0.8	0.2	0.0001
Strategy change during study							
No	7	2	7	0	5	0	
Yes	11	4	6	2	0	1	0.09

## Discussion

In contrast to our previous meta-analysis [1], which was a compilation of conventional meta-analyses, the present network meta-analysis indirectly compared the different treatment principles arranged in a network anchored on single DMARD therapy. The analysis is the first network meta-analysis to use the essential outcome (joint destruction) and to show that different biologic treatments combined with methotrexate may not be superior to treatments with 2–3 DMARDs or 1–2 DMARDs + LDGC (Figure 10). Furthermore the different biologic treatments did not differ from each other. The latter finding confirms the reliability of the analysis, as it is in agreement with previous network meta-analyses using ACR50 as an outcome [9]–[10], [54]–[59], which indicate that TNF inhibitors, tocilizumab and rituximab have similar effects, abatacept is borderline inferior and IL1i is clinically and statistically inferior. Most of these used a Bayesian framework, but one used a statistical method based on Bucher’s design, similar to ours [57]. The outcome of this analysis corresponded to the outcome of the others and ours. A limitation is that the outcomes of the present and previous network meta-analyses are based on indirect data. Therefore doubt can be raised that the treatment arms compared may not be as comparable as randomized treatment arms from one population. This doubt can never be completely eliminated and therefore some reservation concerning the outcomes should be acknowledged. Consequently, the present analysis cannot be considered to be definite evidence that two or more DMARDs prevent structural joint damage to the same degree as a biologic agent combined with methotrexate. The reverse conclusion is also not definite. Therefore confirmation of the present results in direct comparison studies and meta-analyses would be desirable. Recently, a few such studies did confirm that the effect of triple DMARD therapy was comparable with the effect of TNFi plus methotrexate [5]–[7]. These studies, which were published after the date of our final literature search, did not fulfill our inclusion criteria, as they did not use a single DMARD therapy treatment arm. Similar direct comparisons of the other biologic drugs (tocilizumab, abatacept and rituximab) with combination DMARD treatment have not been performed.

Our approach to reduce heterogeneity was successful, as there was no heterogeneity after exclusion of a single study, neither when the studies were analyzed in one group (Figure 2) nor when the treatments were analyzed separately (Figures 4–9). Most within study bias sources (Table 1) were equally distributed across the defined treatment groups (Table 2) and only one of the Cochrane defined bias domains (incomplete outcome data) was dominated by the high risk of bias grade C (26 of 39). Sensitivity analyses of the bias sources, which were unequally distributed in the combination treatment groups (Tables 2 and 3), did not change the results (Figure 12) with the exception TNFi studies with incomplete outcome data (Figure 12, line 9). This bias could inflate the effect of TNFi, but not change the main finding of the study. In general the results were robust. The amount of evidence in the network was significant (Figure 3), the heterogeneity analysis of the study effects was insignificant indicating similar results from study to study (Figure 2) and direct and indirect comparisons were consistent when comparing treatment balanced data. The main reason for the low degree of heterogeneity was probably that all comparisons were anchored on a similar comparator (single DMARD) and that the baseline differences between included populations were moderate. Finally, publication bias (Figure 11), or other possible confounders such as different disease duration , different disease activity at baseline (PARPR), different use of glucocorticoid or treatment strategy change during the treatment period (Table 3) could not explain the similar outcome effects (Figure 12).

A recent study indicated that patients included in newer studies have a lower baseline disease activity than in older studies [60]. This could in theory explain why the effect of the biologics did not exceed the effect of the DMARDs. This theory is in part confirmed by the fact that there was a difference in baseline disease activity between TNFi studies (PARPR  = 1.9%) and triple DMARD studies (PARPR  = 5.2%). However, the sensitivity analyses of studies with high baseline activity versus low baseline activity showed no differences (Figure 12, lines 12–14). Furthermore, the baseline activity of the double DMARD studies did not differ from the baseline activity of the other biologic studies (Table 3). Consequently the different time periods of the different studies could probably not explain the similar effects.

The chosen outcome (joint destruction) is the essential outcome of RA [61]–[62]. Furthermore, the ACR response criteria used in the meta-analyses of biologic studies [9]–[10], [54]–[59] are not available in older DMARD studies. We accepted two different scoring methods as our previous analysis showed concordant results for both methods [1]. This outcome and other outcome measures of RA are mutually dependent. Although joint inflammation and joint destruction are not always linked, several studies have shown that on the average there is a very high association between integrated measures of inflammatory variables (i.e. ESR, CRP, swollen joint count) and the radiographic score, as shown and reviewed previously [63]–[64]. Therefore, the radiographic score is a cumulative measure that not only shows the existing status of the patient, but also reflects the preceding disease course [63]–[64]. The assumption that the radiographic progression sufficiently reflects the outcome of RA is further verified by the fact that network-meta-analyses comparing biologic drugs using ACR response criteria as outcome measure also do not find differences between the different biologic drugs except that the IL1 inhibitor has an inferior effect [9]–[10], [54]–[59].

All approved treatment principles were investigated. The grouping of DMARDs and LDGC was based on the findings of our previous analyses, which showed that these drugs had similar effects [1]. The present study confirms that the effect of LDGC corresponds to the effect of a DMARD (Figure 12, line 1–2). Our assumption of equality between methotrexate, sulfasalazine and leflunomide has recently been verified in an independent review [65], which, however, did not investigate cyclosporine and gold.

In general, our results agree with those of an independent research group [66], which in an analysis of pairwise meta-analyses indicated that DMARD and TNFi/methotrexate combinations had equal efficacy on ACR response, withdrawals for inefficacy, disability and erosive progression.

Because of the high prices of biologics, their cost-effectiveness is a matter of debate [67]. This may be a reason why different official treatment recommendations are not completely concordant.

Our results are not consistent with the European League against Rheumatism (EULAR) recommendations [68], which suggest that in DMARD naive patients, irrespective of the addition of glucocorticoids, DMARD mono therapy rather than combination therapy of DMARDs may be applied followed by switching to another single DMARD or addition of a biologic agent. In contrast to the EULAR guidelines, the American College of Rheumatology (ACR) guidelines does recommend combination DMARD treatment [69]. However, ACR also recommends biologic treatment to subgroups of patients with poor prognostic factors, who have either received single DMARD therapy or never received DMARDs.

A recent analysis concluded that the continued use of placebo arms instead of active arms in the controlled trials of new biologic agents exposed patients in the control arms to possible deterioration [2]. In an accompanying editorial [70], the previous use of placebo was in part defended, but it was also acknowledged that new designs were necessary to reduce the risk of patients in the control arms. In our opinion there is now evidence that combination treatment with at least two DMARDs, one of which could be LDGC, may prevent structural joint damage to the same degree as a biologic agent combined with methotrexate. Therefore future study designers should not seek superiority of the new drug compared with placebo, but should design studies with sufficient power to demonstrate equality with a combination of conventional DMARDs. Biologic agents should, as originally intended, be reserved for patients that are insufficiently treated with a combination of at least two conventional DMARDs.

## Supporting Information

Checklist S1 **PRISMA Checklist.**
(DOC)
